# Identification and Computational Analysis of Novel Pathogenic Variants in Pakistani Families with Diverse Epidermolysis Bullosa Phenotypes

**DOI:** 10.3390/biom11050620

**Published:** 2021-04-22

**Authors:** Fehmida F. Khan, Naima Khan, Sakina Rehman, Amir Ejaz, Uzma Ali, Muhammad Erfan, Zubair M. Ahmed, Muhammad Naeem

**Affiliations:** 1 Medical Genetics Research Laboratory, Department of Biotechnology, Quaid-i-Azam University, Islamabad 45320, Pakistan; fehmidafaridkhan@hotmail.com (F.F.K.); naimakhan89@gmail.com (N.K.); 2 Laboratory of Neurogenetics and Translational Research, University of Maryland School of Medicine, Baltimore, MD 21201, USA; srehman@som.umaryland.edu; 3 Army Medical College, Rawalpindi 46000, Pakistan; amer_ejaz@yahoo.com; 4 Department of Dermatology, Capital Hospital, Islamabad 44000, Pakistan; druzmaali@gmail.com; 5 Department of Dermatology, Federal Government Polyclinic Hospital, Islamabad 44000, Pakistan; erfan_khattak@yahoo.com

**Keywords:** epidermolysis bullosa, whole exome sequencing, *PLEC*, *ITGB4*, EBS-Ogna

## Abstract

Epidermolysis bullosa (EB) includes a group of rare gesnodermatoses that result in blistering and erosions of the skin and mucous membranes. Genetically, pathogenic variants in around 20 genes are known to alter the structural and functional integrity of intraepidermal adhesion and dermo-epidermal anchorage, leading to four different types of EB. Here we report the underlying genetic causes of EB phenotypes segregating in seven large consanguineous families, recruited from different regions of Pakistan. Whole exome sequencing, followed by segregation analysis of candidate variants through Sanger sequencing, identified eight pathogenic variants, including three novel (*ITGB4:* c.1285G>T, and c.3373G>A; *PLEC:* c.1828A>G) and five previously reported variants (*COL7A1:* c.6209G>A, and c.1573C>T; *FERMT1:* c.676insC; *LAMA3:* c.151insG; *LAMB3:* c.1705C>T). All identified variants were either absent or had very low frequencies in the control databases. Our in-silico analyses and 3-dimensional (3D) molecular modeling support the deleterious impact of these variants on the encoded proteins. Intriguingly, we report the first case of a recessively inherited form of rare EBS-Ogna associated with a homozygous variant in the *PLEC* gene. Our study highlights the clinical and genetic diversity of EB in the Pakistani population and expands the mutation spectrum of EB; it could also be useful for prenatal diagnosis and genetic counseling of the affected families.

## 1. Introduction

Epidermolysis bullosa (EB) is a diverse group of rare genodermatoses characterized by moderate to severe fragility of epithelial tissues, with blistering and erosions of the skin [1,2]. Skin fragility, blistering or erosion may also be associated with some or all of the following: nail dystrophy, milia, crusts and scarring [3]. Signs typically appear at or near birth and persist over a lifetime. However, the onset of lesions in some individuals may not appear until adolescence or early adult life. Blistering may improve with increasing age in some variants [4,5]. Epidermolysis bullosa affects all ethnic groups without clear gender bias [6], with an estimated prevalence of about half a million people worldwide, many of whom are children [7].

Based on the level of blistering and target proteins, there are four broad clinical categories: EB simplex (where tissue cleavage occurs within the epidermis); EB junctional (where tissue cleavage occurs within the lamina lucida); EB dystrophica (where tissue cleavage occurs in the sublamina densa) and Kindler syndrome (KS) with a mixed plane of cleavage [8]. However, there are more than 30 distinct subtypes based on clinical heterogeneity, immunofluorescence mapping (IFM) and mutation analysis [9]. The molecular aberrations alter the structural and functional integrity of intraepidermal and dermo-epidermal cohesion, and lead to different forms of EB [1].

As of November 2020, variants in twelve genes have been associated with EB Simplex, eight with EB junctional, and one each for EB dystrophica and Kindler syndrome (Table 1). Clinical evaluation does not allow precise classification of EB in patients with mild or moderate skin fragility [8]. Thus, molecular investigation is extremely important for the comprehensive diagnosis of EB subtypes, disease prognosis, genetic counseling and patient management [8,10]. Next-generation sequencing (NGS) has proven to be successful in the rapid, cost-effective and precise diagnosis of different types of EB [11,12]. In the current study, we investigated seven unrelated Pakistani families affected with EB by employing NGS. Besides reporting eight pathogenic variants, we also present the first case of a recessively inherited form of EBS-Ogna associated with a variant in the *PLEC* gene.

## 2. Material and Methods

### 2.1. Subjects and Clinical Evaluation

In this study, seven unrelated families with a suspected diagnosis of EB were recruited from remote areas of Pakistan. The families were identified through the dermatology departments of local hospitals. Following approval by the Institutional Review Board of Quaid-i-Azam University, Islamabad, and after informed consent from the families, detailed clinical history was taken, and a cutaneous examination was conducted. The diagnosis of EB was based on clinical appearance and a biopsy of the affected skin (where possible). Peripheral blood samples from affected and unaffected individuals, where available, from all families were collected in EDTA vacutainers (Cat# BD366450, BD Vacutainer, Franklin Lakes NJ, USA) and stored at 4 °C. Genomic DNA samples were isolated from whole blood using commercially available kits (Cat# 56304, QIAamp, Qiagen, Valencia, CA, USA), while Oragene saliva kits (Cat#OG500, DNA Genotek Inc., Ottawa, ON, Canada) were used for the 3 affected individuals. All DNA samples were quantified using a Quantus Fluorometer (Cat#E6150, Promega, Madison, WI, USA).

### 2.2. Whole-Exome Sequencing and Segregation Analyses

Whole Exome Sequencing (WES) was performed for the proband of each family. Exome libraries were captured using the SureSelect V5-post kit (Agilent Technologies, Santa Clara, CA, USA). The average sequencing depth of the target regions was 142X for 150 bp paired-end reads, and sequencing was performed using an Illumina HiSeq 4000 (Illumina, San Diego, CA, USA). The resultant reads were mapped to the human reference genome (hg19) using the Burrows–Wheeler alignment (BWA) tool (BWA-mem: http://bio-bwa.sourceforge.net/bwa.shtml; accessed on 13 February 2020). Variant calling was performed using the Genome Analysis Toolkit (GATK 3.v4). The HaplotypeCaller pipeline in standard VCF format and variant alleles were annotated using SnpEff (SnpEff_v4.1).

The filtration criteria of WES included the following steps: (1) the prioritization of variants in candidate genes known to be consistent with EB; (2) the selection of non-synonymous, frame-shift, gain/loss of stop and start codons; (3) the selection of variants with allele frequency of less than 1% in the genome aggregation database (gnomAD) (https://gnomad.broadinstitute.org; accessed on 1 September 2020); and (4) the prediction of variant pathogenicity on the bases of PolyPhen2 and SIFT scores. The candidate variants were validated in the probands and their segregation in families by Sanger Sequencing.

### 2.3. Protein Modeling and Bioinformatic Analysis

Clustal omega (https://www.ebi.ac.uk/Tools/msa/clustalo/; accessed on 23 October 2020) multiple sequence alignment was used to appraise the evolutionary conservation of the identified variants. Polyphen-2 (http://genetics.bwh.harvard.edu/pph2/; accessed on 23 October 2020), SIFT (https://sift.bii.a-star.edu.sg/www/SIFT_seq_submit2.html; accessed on 23 October 2020) and the combined annotation-dependent depletion score (version 1.5; https://cadd.gs.washington.edu/score/; accessed on 23 October 2020) were used to evaluate the impact of the identified variants on the encoded proteins. The VarSome (https://varsome.com/; accessed on 24 October 2020) online tool was used for the prediction of pathogenicity of EB-associated variants according to the American College of Medical Genetics and Genomics (ACMG) guidelines. To further evaluate the impact of the variants on secondary structure, 3D protein models were generated through the I-Tasser server (https://zhanglab.ccmb.med.umich.edu/I-TASSER/; accessed on 23 October 2020) and analyzed through the PyMOL system (https://pymol.org/2/; accessed on 23 October 2020). Finally, the stereochemistry and validity of the constructed 3D protein structures were assessed using PDBsum (http://www.ebi.ac.uk/thornton-srv/databases/cgi-bin/pdbsum/GetPage.pl?pdbcode=index.html; accessed on 23 October 2020) and Ramachandran plots.

## 3. Results

After IRB approval and informed consent, we enrolled seven consanguineous families from the dermatology clinics of hospitals in different regions of Pakistan. According to the family medical histories, all affected individuals had skin problems from their childhood. We clinically examined nine patients from these families to characterize their phenotypes, and performed whole exome sequencing (WES) to decipher the underlying disease-causing variants.

### 3.1. Family 1: JEB Laryngo-Onycho-Cutaneous Syndrome

**Figure 1:**
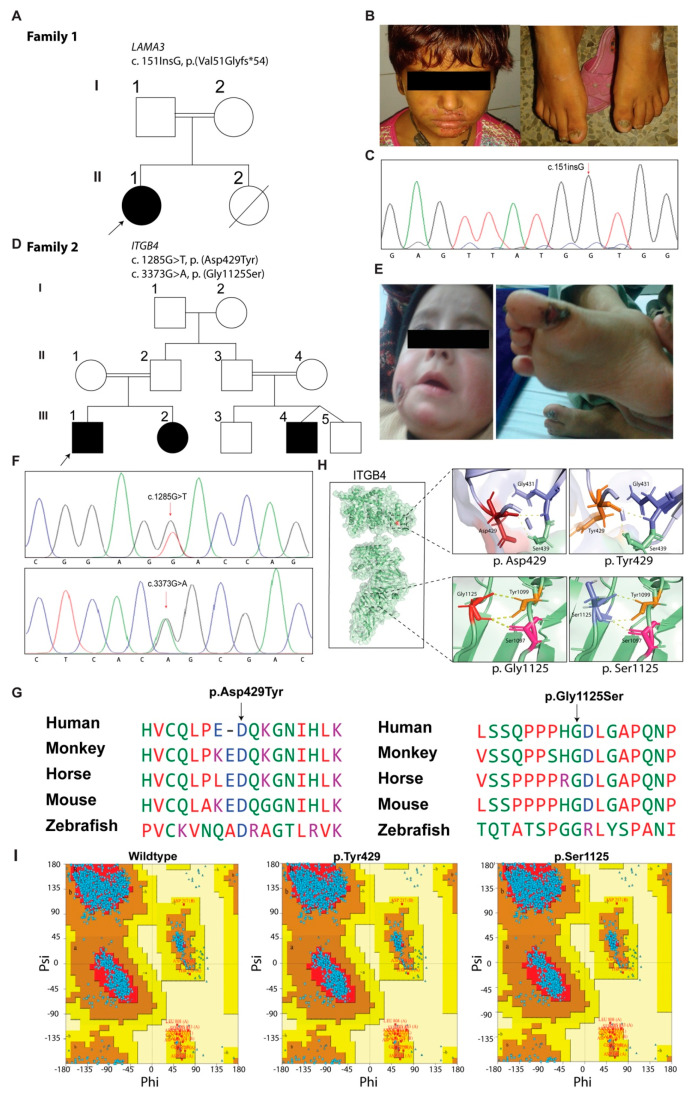
EB phenotypes in families 1 and 2 are associated with pathogenic variants of *LAMA3* and *ITGB3*, respectively. (**A**) Pedigree of family 1. Filled and empty symbols represent affected and unaffected individuals, respectively. Double lines indicate consanguineous marriages. The arrow points to the proband. A symbol with a line through it indicates a deceased individual. (**B**) Clinical manifestations of the proband (II:1), showing facial erosion and thickened nails. (**C**) Chromatogram showing homozygosity for the identified variant (c.151insG) in the proband. (**D**) Family 2 pedigree with three affected individuals in two sibships. (**E**) Hemorrhagic crust on the blister site after rupturing bullae and nail dystrophy; hemorrhagic crust on the cheek of the patient (III:2) after rupturing bullae. (**F**) Chromatogram showing homozygosity and heterozygosity for both the identified variants, c.1285G>T and c.3373G>A), of *ITGB4*. (**G**) Multiple sequence alignment of ITGB4 revealed the evolutionary conservation of p.Asp429 and p.Gly1125 residues. (**H**) 3D Protein modeling of the ITGB4 protein. Wild-type and mutated residues at position 429 are shown in maroon and orange, while at position 1125 they are shown in red and purple, respectively. Wild-type and mutated residues of ITGB4 are found at positions 429 and 1125, although they have no apparent impact on the ionic interactions but might alter the protein secondary structure due to size differences. (**I)** Ramachandran plots for the wild-type and mutated residue of ITGB4 reveal that 88.5% of the residues are present in the allowed (acceptable geometries) region, suggesting there is no significant impact on the stereochemistry and geometry of the encoded protein due to the ITGB4 variants found in family 2.

The proband (II:1) in family 1 is a seven-year-old girl born to a consanguineous Punjabi family (Figure 1A). She developed facial erosion (Figure 1B), and subungual granulation tissue along with severe hoarseness of voice at the age of six months. The formation of granulation tissue under the toenails led to shedding of the nails, followed by fingernail involvement. The nails were curved and thickened, and regrowth was slow (Figure 1B). She also had bilateral conjunctival masses and tooth anomalies. Her clinical presentation is consistent with the cardinal features of juvenile EB (JEB) laryngo-onycho-cutaneous (LOC) syndrome. Her younger sibling (II:2), who died at the age of 18 months due to airway obstruction, had no symptoms of LOC syndrome. To investigate the disease-causing variant, we performed exome sequencing on the proband DNA sample, which revealed a single nucleotide insertion mutation (c.151insG) in exon 39 of *LAMA3*, a commonly occurring variant in LOC cases. Sanger sequencing confirmed the homozygosity in the proband (II:1), whereas her parents (I:1 and I:2) were obligate carriers (Figure 1C).

### 3.2. Family 2: JEB without Pylori Atresia

Family 2 had two affected individuals (III:1 and III:2) born to consanguineous parents (Figure 1D). According to the parents, skin blistering on the hands of the proband (III:1) was first noticed four hours after his birth, followed by sparsely occurring blisters on his outer ears, feet and cheek (Figure 1E). Blisters and erosions healed without scarring, atrophy or milia and deteriorated mostly both by extensive walking and during the summer. Enamel hypoplasia, and nail dystrophy affecting all fingernails and toenails (Figure 1E), were present at the time of study. Pyloric atresia and urologic complications were not documented. A similar history was noted in his younger sibling (III:2).

WES, followed by the analysis of data according to the filtration criteria given in the material and methods section, narrowed down the candidate nucleotide changes to four, including two heterozygous variants: c.1285G>T (p.(Asp429Tyr)) in exon 11 and c.3373G>A (p.(Gly1125Ser)) in exon 28 of *ITGB4* (Figure 1F). Segregation analysis through Sanger sequencing confirmed the inheritance of c.3373G>A variant from the mother, while the father transmitted the c.1285G>T allele, resulting in a compound heterozygous state in both the affected individuals. Both variants were present in the evolutionarily conserved regions of the encoded protein (Figure 1G). These variants were absent or had very low frequencies in the gnomAD database, had high CADD scores, were predicted as damaging by in silico algorithms, and were categorized as “pathogenic” or “likely pathogenic”, respectively, according to the ACMG classification criteria (Table 2).

Phyre2 software-based modeling of ITGB4 revealed that p.Asp429Tyr and p.Gly1125Ser variants do not have an apparent impact on the hydrogen bonding with neighboring residues (Figure 1H), but might alter the protein’s secondary structure due to size differences in the wild-type and mutant residues. We also used the predicted models of both wild-type and mutated proteins to further evaluate the bond angles using a Ramachandran plot, a tool to assess the stereochemistry and geometry of the protein and to assess if any of the geometries are in the forbidden electrostatically unfavored regions. Our analysis did not reveal any difference, as 88.5% of amino acids were in the allowed region for both cases (Figure 1I). Finally, the Phi-Psi distribution Z-scores for both wild-type and mutant ITGB4 were (−0.50 ± 0.08) and (−0.37 ± 0.08), respectively, showing that the Ramachandran distribution is in a comparable range. These molecular predictions suggested that the ITGB4 variants identified in family 2 are likely impairing the function leading to the EB phenotype, without any major effect on the structure of the encoded proteins.

### 3.3. Family 3: EBS-Ogna Type

**Figure 2:**
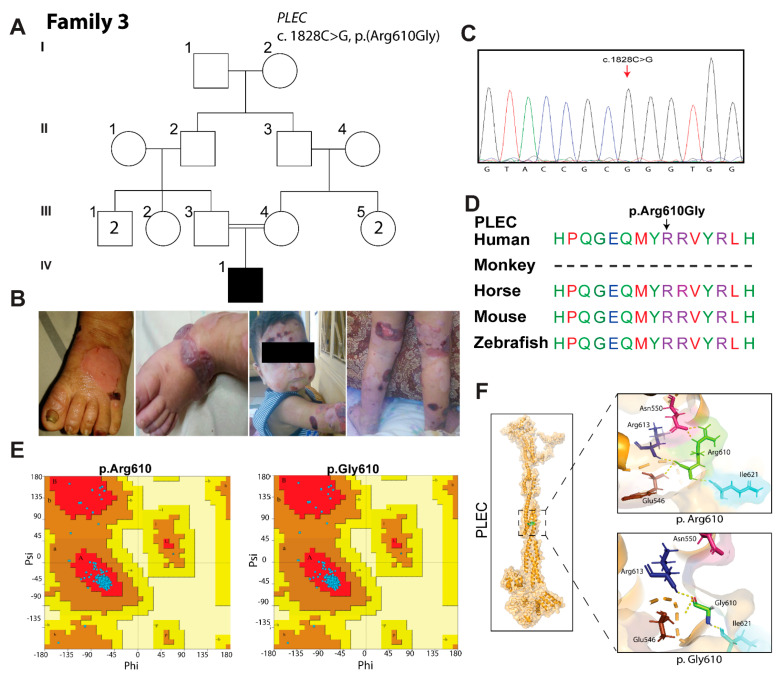
EB phenotype in family 3 is associated with pathogenic variant of *PLEC.* (**A**) Pedigree of family 3. (**B**) Clinical features of enrolled patients showing cutaneous erosion, hemorrhagic crust and hypopigmented macules after rupturing of blisters, and nail dystrophy. (**C**) Chromatogram showing homozygosity in the patient (IV:1). (**D**) Clustal omega multiple sequence alignment showing conservation of arginine residue across different species (**E**) Ramachandran plot of wild-type and mutated protein reveals 98.6% residues in the allowed region; there is thus no significant impact on the stereochemistry and geometry of the encoded protein due to the p.Arg610Gly change. (**F**) 3D protein modeling of PLEC revealed a loss of ionic interactions (yellow dotted lines) with other residues in the core of the protein, due to the p.Arg610Gly variant.

This was a single case of a 2-year-old boy (IV:1) born to unaffected consanguineous parents and without any prior history of EB in the family (Figure 2A). He developed generalized hemorrhagic blistering when he was three days old. Cutaneous erosion, hemorrhagic crust and hypopigmented macules developed after rupturing of the blisters (Figure 2B). He had dystrophy of both fingernails and toenails (Figure 2B) without mucosal involvement. Parents reported the severe blistering of the patient in the summer. There was no evidence of muscular dystrophy or pyloric atresia.

WES analysis of the proband (IV:1) DNA sample revealed the homozygous missense variant c.1828A>G in exon 13 of the *PLEC* gene, which is predicted to substitute an arginine residue at position 610 for glycine (p.Arg610Gly). Sanger sequencing confirmed the homozygosity of the c.1828A>G variant in the proband (Figure 2C), while the parents were obligate carriers, and had no EB symptoms. Dominantly inherited variants of *PLEC* are associated with the EBS-Ogna type. The p.(Arg610Gly) variant represents the first recessively inherited variant of *PLEC* causing the EBS-Ogna type. Several in silico algorithms predicted the damaging impact of the p.(Arg610Gly) variant on the encoded protein, and it was categorized as pathogenic according to the ACMG classification (Table 2). Clustal omega multiple sequence alignment revealed a high evolutionary conservation of arginine residue at position 610 (Figure 2D). Phyre2-based 3D protein modeling of the PLEC revealed loss of ionic interactions (yellow dotted lines) with other residues in the core of the protein due to the p.Arg610Gly variant (Figure 2F). The Ramachandran plot did not depict any significant differences in the bond angles and Phi-Psi distribution (0.76 ± 0.40) due to the p.Arg610Gly variant (Figure 2F).

### 3.4. Family 4: Dominant EB Dystrophica, Pruriginosa Type

**Figure 3:**
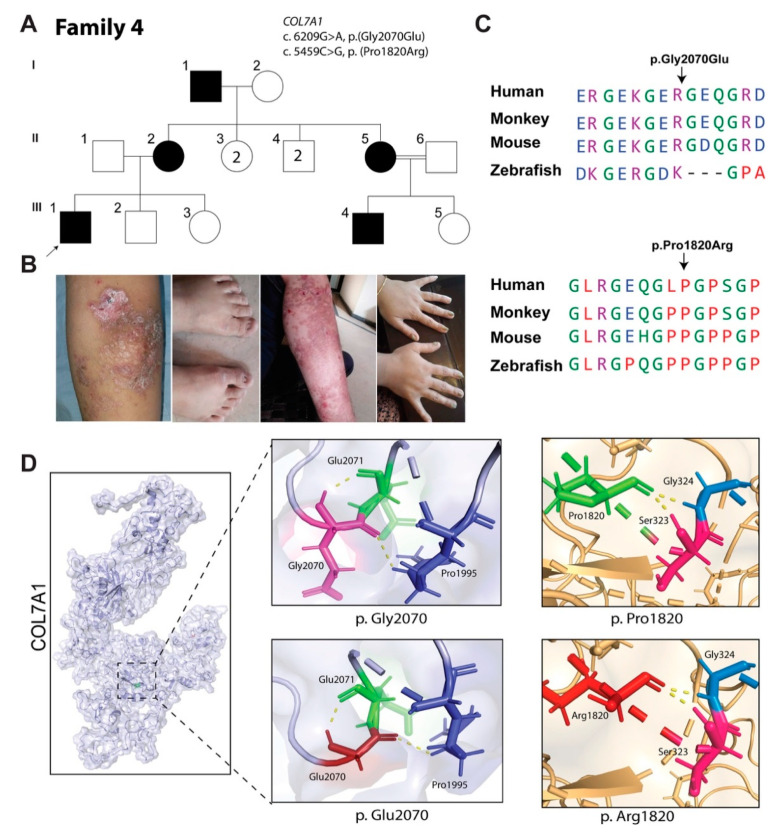
EB phenotype in family 4 is associated with compound heterozygous variants of *COL7A1*. (**A**) Pedigree of family 4. (**B**) Clinical features of enrolled patients showing erosions and scarring with pruritic eruption affecting shins, dystrophy of fingernails, erosion and crust on elbow, and nail dystrophy of the hands in the patient (II:1). (**C**) Alignment of COL7A1 across different vertebral species. (**D**) Predicted 3D protein structure of COL7A1. Wild-type residue at position 2070 is shown in purple and red, while at position 1820, they are shown in green and red, respectively; apparently, both mutations are not affecting ionic interactions but can impact protein folding and secondary structure.

The proband in family 4 is a 12-year-old boy (III:1; Figure 3A) with a history of blistering, erosions and scarring, with pruritic eruption affecting his shins, feet, knees and elbows. He also had dystrophy of the fingernails and anonychia of the toenails (Figure 3B). The first blisters appeared on his feet six days after birth. His mother (II:1) and grandfather (I:1) were also affected with the same phenotype, but their blistering and pruritic eruption disappeared after the second decade of their life. Now, both the mother and grandfather have only the symptoms of hyperkeratotic fingernails, anonychia of toenails and scarring on the shins. There was no associated mucosal involvement in any of the affected individuals. The evaluating clinician classified the phenotype segregation in family 4 as a dominantly inherited form of EB dystrophica, pruriginosa type.

WES of the proband sample revealed two heterozygous variants, c.6209G>A (p.(Gly2070Glu)) and c.5459C>G (p.(Pro1820Arg)) in *COL7A1*, and another heterozygous variant (c.2654C>A; p.(Pro885His)) in *ITGA3*. Sanger sequencing revealed the segregation of both *COL7A1* variants with EB dystrophica phenotype in the family (data not shown). The c.6209G>A variant has been previously reported in EB dystrophica patients; however, the c.5459C>G is novel. Both variants are categorized as “pathogenic” or “likely pathogenic”, respectively, according to ACMG classification criteria (Table 2), and are inherited in cis-configuration from the grandfather. Multiple sequence alignment revealed a high conservation of both mutated residues during evolution (Figure 3C). 3D protein structure prediction did not show any difference in the hydrogen bond angles between wild-type and mutant residues (Figure 3D), but the size and charge differences might impact the protein folding.

### 3.5. Family 5: Kindler Syndrome

**Figure 4:**
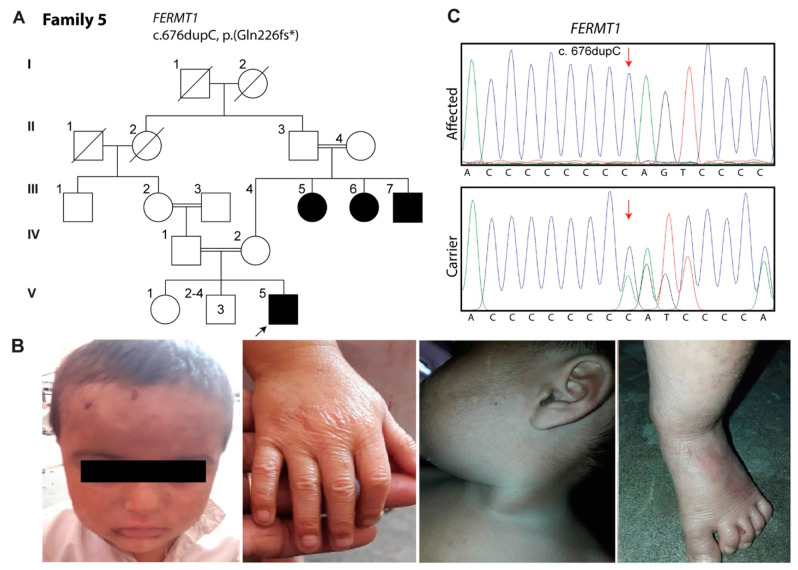
EB phenotype in family 5 is associated with pathogenic variants of *FERMT1*. (**A**) pedigree of family 5, (**B**) skin atrophy of hands and feet, absence of poikiloderma, hypopigmentation on the forehead, (**C**) chromatogram showing heterozygosity for identified mutation (c.676dupC) in parents and homozygosity in the patient (V:5). The asterisk indicates premature termination codon.

The proband in family 5 is a 2-year-old boy (V:5; Figure 4A). According to his parents, the first manifestation was noted shortly after birth in the form of blisters on his hands followed by skin atrophy, particularly on the dorsal aspect of the hands and feet. He had hypopigmentation on the forehead and hyperpigmentation on the legs (Figure 4B). There were no symptoms of poikiloderma, gingivitis, periodontitis, esophageal or urogenital stenosis, or of gastrointestinal complications at the time of examinations.

WES revealed a homozygous frameshift variant c.676dupC (p.(Gln226fs*16)) located in exon 5 of the *FERMT1* gene in the proband. Sanger sequencing confirmed the homozygosity of the variant in the proband (Figure 4C), while the parents were obligate carriers, and the participating unaffected sibling was of a homozygous wild type.

### 3.6. Family 6: Herlitz JEB

**Figure 5:**
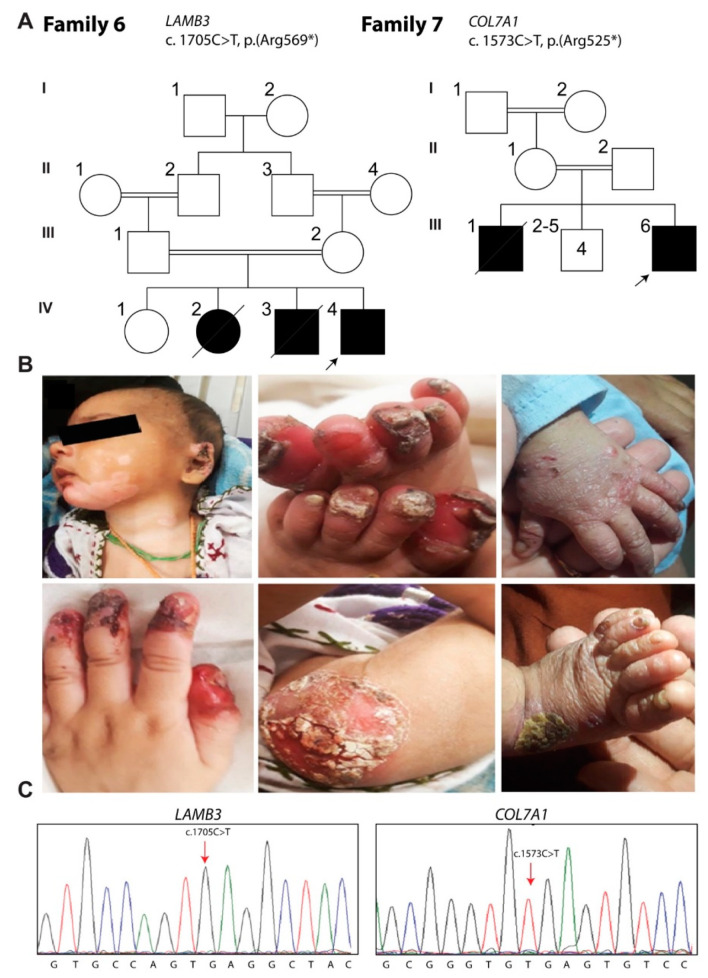
The EB phenotype in family 6 and 7 is associated with pathogenic variants of *LAMB3* and *COL7A1,* respectively. (**A**) Pedigrees of family 6 and 7. (**B**) Hypopigmented patches on front of upper chest, neck and chin with crust plaque on the left ear, erythematous and ulcerated lesions with nail dystrophy, hyperkeratotic annular plaque, demarcated erosions with erythematous base, milia on both hands and onychogryphosis, and a single yellowish crusted lesion along with onychogryphosis and anonychia. (**C**) Chromatogram showing homozygosity of identified variants in the patients: (c.C1705T) in IV:4 and (c.C1573T) in III:6. The asterisk indicates premature termination codon.

A family from Islamabad, Pakistan, affected with Herlitz JEB, was included in the study. The proband (IV:4) was born to the phenotypically normal couple (III:1 and III:2), and the inheritance pattern is consistent with an autosomal recessive transmission of the disease-causing variant (Figure 5A). The clinical symptoms in the proband were first observed seven days after birth, including blistering all over the body, mainly on his hands and feet. He also had hypopigmented patches involving the front of his upper chest, neck and chin, with crust plaque on the left ear (Figure 5B; top panels). Erythematous and ulcerated lesions of the digits of both feet and hands, along with nail dystrophy, were noted at the time of clinical evaluation. Hyperkeratotic annular plaque was present on the trauma-prone area of the knee (Figure 5B; down panels). His elder brother and sister died four months after birth due to the same clinical features, according to his parents.

In family 6, WES revealed a homozygous nonsense variant c.1705C>T (p.(Arg569*)) located in exon 13 of *LAMB3* in the proband’s DNA sample. Sanger sequencing further confirmed the homozygosity for the c.1705C>T variant in the proband (Figure 5C), while both parents (III:1 and III:2) were heterozygous. This variant has been previously reported in unrelated patients with Herlitz JEB, and thus may represent a mutation hotspot.

### 3.7. Family 7: EB Dystrophica

The EB dystrophica proband of family 7 was ascertained from Islamabad Capital Territory, Pakistan. The consanguineous family had two affected offspring (III:1 and III:6) from normal parents (Figure 5A). The patient was 5 months old (III:6) at the time of examination. Shortly after birth, he developed blisters on his hands and heels, followed by blistering all over his body, mainly on the hands and feet. He had well-demarcated erosions with an erythematous base present on the side of the bridge of his nose. He developed blisters in the mouth after using the feeder. Numerous milia were present on both hands, with nail dystrophy in the form of onychogryphosis (Figure 5B; top panels). A single yellowish crusted lesion was present on the ventral surface of the foot in proximity to medical malleolus, along with onychogryphosis and anonychia (Figure 5B; bottom panels). His elder brother died eight days after his birth, due to the same clinical features.

WES of the proband (III:6) revealed a homozygous nonsense variant c.1573C>T (p.Arg525*) in exon 12 of *COL7A1*, which was further confirmed by Sanger sequencing (Figure 5C). Both parents (II:1 and II:2) and the unaffected sibling (III:2) were heterozygous for the c.1573C>T variant. This variant has been previously reported in patients with EB dystrophica.

## 4. Discussion

There are four main types of EB based on the level of skin damage at the ultrastructural level, and more than 30 subtypes based on clinical outcomes, inheritance and molecular aberrations [9]. Clinically, it is difficult to accurately diagnose the type and subtype of EB especially in newborns, which is vital for prognostics, genetic counseling and patient management [13]. Next-generation sequencing (NGS) could potentially diagnose the type and subtype of EB in one sample [11,14]. Recently, Lucky et al. reported the use of an NGS-based diagnostic assay called EBSEQ that allows simultaneous analysis of genes known to be involved in epidermolysis bullosa pathogenicity [12]. These new technologies omit the use of methods required for the selection of the gene to be sequenced, such as direct immunofluorescence mapping (DIF) or transmission electron microscopy (TEM) [15,16]. In this study, we used WES to directly identify the causative gene of epidermolysis bullosa in different Pakistani families (Families 1–7) and found disease-associated variants in the known EB genes.

In family 1, we found a c.151insG variant of *LAMA3* segregating with the laryngo-onycho-cutaneous syndrome (LOC) phenotype. LOC syndrome is classified as a rare subtype of JEB, mainly caused by the same founder mutation (c.151insG) in exon 39 of the *LAMA3* gene [1,17,18]. *LAMA3* encodes a subunit of a protein called laminin 332, which functions as a supramolecular bridge between the epidermis’s basal keratinocytes and the underlying dermis. The loss of the laminin-a3a N-terminal region (226 amino acids) causes aberrant keratinocyte mesenchymal signaling, underlying the excessive response of granulation tissue. Mostly, LOC syndrome patients expire due to airway obstruction, caused by the accumulation of granulation tissue in the larynx [17].

In family 2, we identified a novel compound heterozygous mutation c.1285G>T (p.Asp429Tyr); c.3373G>A (p.Gly1125Ser) in the *ITGB4 gene*. The integrin α6β4 is predominantly expressed in skin, gastrointestinal and urinary epithelia [19,20]. Pathogenic variants of the *ITGB4* gene can cause lethal and non-lethal forms of JEB, with a predominance of nonsense alleles in the lethal forms, whereas missense, compound heterozygous and some nonsense mutations are more common in the non-lethal JEB [19]. Both the patients of family 2 (III:1 and III:2) with compound heterozygous missense variants had mild blistering on their hands, feet, cheek, and outer ears, without the involvement of pyloric atresia and urinary tract complications. Some studies have suggested that pyloric atresia is not necessarily a characteristic of integrin-associated JEB, in contrast to urologic problems, which occur frequently [19,20,21]. Enamel defects and nail dystrophy, affecting all fingernails and toenails, were present in both patients, consistent with previously reported cases of JEB without pyloric atresia [22].

Plectin, encoded by the *PLEC* gene, functions as a cytolinker which is expressed in a wide variety of tissues including the skin, mucous membranes, gut, and muscle tissue [23,24]. As is consistent with its multiple binding partners, defects in the *PLEC* gene cause three different types of basal EBS: EBS with pyloric atresia, EBS with muscular dystrophy, and EBS-Ogna with only skin fragility [25]. The proband (IV:1) of family 3 had generalized hemorrhagic blistering followed by cutaneous erosion, hemorrhagic crust and hypopigmented macules, and nail dystrophy of both hands and feet. He had not demonstrated clinical signs of pyloric atresia and muscular dystrophy, as is consistent with previous cases of EBS-Ogna [26,27]. In the proband, WES identified a homozygous mutation c.1828A>G in exon 13 of the *PLEC* gene. Heterozygosity of both parents for the identified variant with normal phenotype supported the autosomal recessive mode of inheritance, which is not reported in previous cases of EBS-Ogna [26,27,28]. So far, only three pathogenic variants (c.5998C>T, p.(Arg2000Trp); c.8668A>T, p.(Thr2890Ser); c.10579C>T, p.(Arg3527Cys)) in the heterozygous state are reported in EBS-Ogna cases [26,27,28]. The recessively inherited variant c.1828A>G, found in family 3, is predicted to convert an arginine residue to glycine residue on position 610 of the plectin protein (p.Arg610Gly), which may affect the globular plakin domain of N-terminal harboring binding sites for integrin α6β4 [29] and type XVII collagen [30]. The identification of a recessive form of the *PLEC* variant expands the repertoire of the known genetic causes of EBS-Ogna and the phenotypic spectrum of *PLEC* alleles. Intriguingly, the parents’ heterozygous states for the c.1828A>G (p.(Arg610Gly)) variant are normal, suggesting that different pathologic mechanisms might be involved in the dominant and recessive forms of PLEC-associated EBS-Ogna.

Family 4 was diagnosed with epidermolysis bullosa pruriginosa, which is also a rare subtype of DEB caused by variants in the *COL7A1* gene, the key structural protein of anchoring fibrils at the dermo-epidermal junction [31]. The proband (12 years old) of family 4 manifests severe pruritus, lichenified plaques or prurigo-like lesions, trauma-induced blistering, scarring and nail dystrophy, whereas all these symptoms disappeared in his mother and grandfather, except for scarring on lower legs and nail dystrophy, after their second decade of life. Identical variants of the *COL7A1* gene can cause both EB dystrophica and EB pruriginosa, either in dominant or recessive inheritance [32]. Our study expands the allelic spectrum of *COL7A1* disease-causing variants.

Kindler syndrome (KS) is a rare autosomal recessive genodermatosis, classified as a less severe subtype of epidermolysis bullosa [1]. It is characterized by congenital blistering on trauma-induced sites and the later development of poikiloderma, skin atrophy, varying degrees of photosensitivity, and periodontal abnormalities [33]. Clinically, the proband (V:5) of family 5 was initially diagnosed as an EBS patient, but WES identified a homozygous insertion (c.676insC) in exon 5 of the *FERMT1* gene which is responsible for KS [34]. This mutation was previously reported in several unrelated Pakistani KS families [35,36,37]. Thus, genetic studies facilitated a complete clinical diagnosis and disease management.

In the proband (IV:4) of family 6, WES identified a homozygous nonsense variant, c.1705C>T (p.Arg569*), in exon 13 of the *LAMB3* gene, which was previously reported with Herlitz JEB in a Caucasian family. The cytosine to thymine transitions might occur persistently in this gene due to cytosine hypermutability in CpG dinucleotide sequences, potentially resulting in the JEB phenotype [38]. The majority of reported variants in *LAMB3* are predicted to result in premature stop codons, leading to nonsense-mediated decay of mRNA and the absence of protein or synthesis of truncated unstable polypeptides [39,40].

In family 7, a homozygous nonsense variant c.1573C>T in exon 12 (p.Arg525*) of the *COL7A1* gene was identified by WES. This variant is previously reported to cause generalized severe EB dystrophica [41], which is characterized by generalized blistering from birth, followed by extensive scarring and pseudosyndactyly [42]. The proband (III:6) of family 7 manifested generalized blistering shortly after birth, without pseudosyndactyly. Mucous membrane involvement is reported in these patients, which mainly causes problems in oral feeding [1], as observed in our patient when fed by a feeder. His elder brother died a few days after birth, which is consistent with previous reports that these patients rarely survive beyond the age of 30 [43]).

Collectively, our findings highlight the complexity of the clinical characteristics and genetics of EB. Our findings will likely help in its complete diagnosis, family counseling, and disease management.

## Figures and Tables

**Table 1:** Genes involved in different types of EB.

EB Type	Genes
Epidermolysis bullosa simplex	*KRT5, KRT14, JUP, DSP, DST, EXPH5, PLEC, PKP1, TGM5, ITGA6, ITGB4, KLHL24*
Epidermolysis bullosa junctional	*LAMA3, LAMB3, LAMC2, ITGA6, ITGB4, ITGA3, COL 17A1, CD151*
Epidermolysis bullosa dystrophica	*COL7A1*
Kindler syndrome	*FERMT1*

**Table 2:** Epidermolysis bullosa-causing genes, identified variants and their ACMG classification.

Gene	cDNA Change	Protein Change	CADD	GnomAD	Polyphen2	SIFT	ACMG Classification (Criteria Used)
*ITGB4*	c.1285G>T	p.(Asp429Tyr)	32	0	Damaging	Damaging	Pathogenic (PVS1, PM2, PP3, PP5)
	c.3373G>G	p.(Gly1125Ser)	21.4	0.000127	Probably damaging	Damaging	Likely pathogenic (PP2, PP3)
*PLEC*	c.1828C>G	p.(Arg610Gly)	25.4	0	Possibly damaging	Damaging	Likely pathogenic (PS3, PP2, PP3)
*COL7A1*	c.6209G>A	p.(Gly2070Glu)	28.7	0	Damaging	Damaging	Pathogenic (PVS1, PM2, PP3, PP5)
	c.5459C>G	p.(Pro1820Arg)	17.8	0.000256	Damaging	Tolerated	Likely pathogenic (PP2, PP3)

CADD: combined annotation dependent depletion, https://cadd.gs.washington.edu/; gnomAD: genome aggregation database, https://gnomad.broadinstitute.org; PVS1: pathogenic, very strong (null variant (nonsense, frameshift, canonical ±1 or 2 splice sites, initiation codon, single or multiexon deletion) in a gene where LOF is a known mechanism of disease))**;** PM2: pathogenic moderate 2 (absent from controls (or at extremely low frequency if recessive) in Exome Sequencing Project, 1000 Genomes Project, or exome aggregation consortium); PP3: pathogenic supporting 3 (multiple lines of computational evidence support a deleterious effect on the gene or gene product (conservation, evolutionary, splicing impact, etc.)); PP5: pathogenic supporting 5 (reputable source recently reports variant as pathogenic, but the evidence is not available to the laboratory to perform an independent evaluation); BP1: benign supporting 1 (missense variant in a gene for which primarily truncating variants are known to cause disease); BP4: benign supporting 4 (a benign computational verdict because of 1 benign prediction from GERP vs. no pathogenic predictions).

## Data Availability

The whole exome sequencing data generated during the current study will be available through the dbGAP (https://www.ncbi.nlm.nih.gov/gap/ accessed on 23 October 2020) repository.
